# Decline in Decreased Cephalosporin Susceptibility and Increase in Azithromycin Resistance in *Neisseria gonorrhoeae*, Canada

**DOI:** 10.3201/eid2201.151247

**Published:** 2016-01

**Authors:** I. Martin, P. Sawatzky, G. Liu, V Allen, B. Lefebvre, L. Hoang, S. Drews, G. Horsman, J. Wylie, D. Haldane, R. Garceau, S. Ratnam, T. Wong, C. Archibald, M.R. Mulvey

**Affiliations:** Public Health Agency of Canada, Winnipeg, Manitoba, Canada (I. Martin, P. Sawatzky, G. Liu, M.R. Mulvey);; Public Health Ontario Laboratories, Toronto, Ontario, Canada (V. Allen);; Laboratoire de Santé Publique du Québec, Québec, Canada (B. Lefebvre);; British Columbia Centre for Disease Control, Vancouver, British Columbia, Canada (L. Hoang);; Provincial Laboratory for Public Health, Edmonton, Alberta, Canada (S. Drews);; Saskatchewan Disease Control Laboratory, Regina, Saskatchewan, Canada (G. Horsman);; Cadham Provincial Laboratory, Winnipeg (J. Wylie);; Queen Elizabeth II Health Sciences Centre, Halifax, Nova Scotia, Canada (D. Haldane);; Dr. G.L. Dumont Hospital, Moncton, New Brunswick, Canada (R. Garceau);; Newfoundland and Labrador Public Health Laboratory, St. John’s, Newfoundland, Canada (S. Ratnam);; Public Health Agency of Canada, Ottawa, Ontario, Canada (T. Wong, C. Archibald)

**Keywords:** *Neisseria gonorrhoeae*, gonorrhoea, gonorrhea, antimicrobial resistance, rates, bacteria, cephalosporins, susceptibility, azithromycin, Canada

## Abstract

Antimicrobial resistance profiles were determined for *Neisseria gonorrhoeae* strains isolated in Canada during 2010–2014. The proportion of isolates with decreased susceptibility to cephalosporins declined significantly between 2011 and 2014, whereas azithromycin resistance increased significantly during that period. Continued surveillance of antimicrobial drug susceptibilities is imperative to inform treatment guidelines.

Gonorrhea, caused by *Neisseria gonorrhoeae*, is the second most commonly reported sexually transmitted infection in Canada; ≈13,000 cases occur yearly, and rates have increased from 20.1 cases/100,000 population in 2000 to 39.2 cases/100,000 in 2013 (*1*). The infection is also a global public health threat, with ≈106 million cases/year occurring worldwide (*2*). Gonococci have acquired resistance to many antimicrobial agents used for treatment (*3*), however, which makes it imperative to conduct surveillance programs so appropriate treatment recommendations can be determined. In 2011, the increases in MICs of cephalosporins prompted the authors of the Canadian Sexually Transmitted Infections Guidelines to update the recommended gonorrhea treatment from a single antimicrobial drug to combination therapy with ceftriaxone (250 mg intramuscularly) and azithromycin (1 g orally in a single dose) as the first-line treatment for uncomplicated anogenital and pharyngeal *N. gonorrhoeae* infections in adults (*4*). We analyzed antimicrobial drug susceptibility levels of *N. gonorrhoeae* to cephalosporins and azithromycin in Canada since the recommended treatments were updated in 2011.

## The Study

The National Microbiology Laboratory (NML) in Winnipeg, Manitoba, Canada, has conducted ongoing monitoring of antimicrobial drug susceptibilities in *N. gonorrhoeae* isolates since 1985. Isolates are submitted to NML by provincial laboratories when they identify a resistant isolate or by laboratories that do not conduct antimicrobial susceptibility testing. To determine the proportion of antimicrobial drug resistance, we used the total number of isolates identified in each province as the denominator.

Antimicrobial drug susceptibilities of *N. gonorrhoeae* to ceftriaxone, azithromycin, and cefixime (Sigma-Aldrich, Oakville, Ontario, Canada) were determined by using agar dilution as previously described (*5*,*6*). MIC breakpoints used were the following: decreased susceptibility to cefixime, MIC >0.25 mg/L; decreased susceptibility to ceftriaxone, MIC >0.125 mg/L (*2*); resistance to azithromycin, MIC >2.0 mg/L (*7*). For controls, we used *N. gonorrhoeae* reference cultures ATCC49226, WHOF, WHOG, WHOK, and WHOP. Statistical analysis was determined by using EpiCalc 2000 version 1.02 (http://www.brixtonhealth.com/epicalc.html). A 2 × 2 χ^2^ test was used to compare proportions of resistance per year to identify significant differences between years (p values calculated with 99% CI).

From 2010 through 2014, ≈59,400 cases of *N. gonorrhoeae* infection were reported in Canada; 16,370 (≈28%) were diagnosed by culture. Provincial public health laboratories submitted 6,728 isolates to NML for testing (2010 = 1,235; 2011 = 1,173; 2012 = 1,035; 2013 = 1,184; 2014 = 2,101). Sex and age data of patients were available for 6,468 (96.1%) isolates. Of these, 5,221 (80.7%) were from male patients (mean age 32.6 years; range <1–83 years) and 1,247 (19.3%) were from female patients (mean age 27.9 years; range <1–83 years). Source specimens included urethral (n = 2,320), rectal (n = 981), pharyngeal (n = 592), cervical (n = 365), vaginal (n = 154), and other sources (n = 85); sources for 2,231 isolates were not given. The sexual orientation of patients and information on cases of treatment failure were not available.

In 2010, a total of 98 (3.3%) of 2,970 isolates had decreased susceptibility to cefixime. This proportion increased to 4.2% (140/3,360) in 2011, and then decreased significantly to 1.1% (42/3,809; p<0.001) in 2014 (Figure). Similarly, decreased ceftriaxone susceptibility was 7.3% (218/2,970) in 2010 and declined to 2.7% (102/3,809; p<0.001) by 2014 (Figure).

**Figure:**
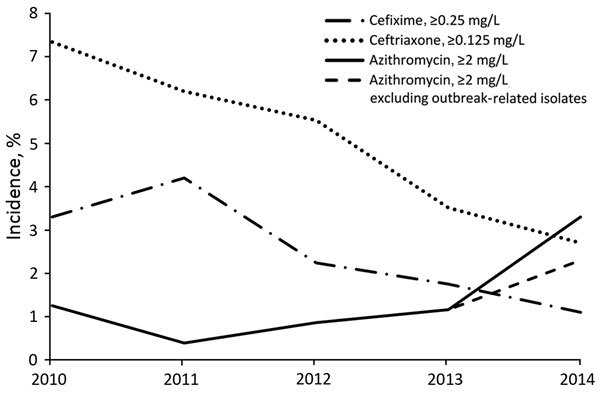
MICs for *Neisseria gonorrhoeae* isolates with decreased susceptibility to cefixime and ceftriaxone and resistance to azithromycin, Canada, 2010–2014. Percentages are based on the total number of isolates tested nationally per year: 2010 = 2,970; 2011 = 3,360; 2012 = 3,036; 2013 = 3,195; 2014 = 3,809.

Before 2010, the proportion of azithromycin resistance was <0.4% (data not shown). Azithromycin resistance increased to 1.2% (37/2,970) in 2010 and then decreased to 0.4% (13/3,360) in 2011. From 2011 to 2014, azithromycin resistance increased significantly to 3.3% (127/3,809; p<0.001). Thirty-eight isolates were identified as part of an outbreak in 1 province. When the outbreak-related isolates were excluded, 2.3% of all the isolates were azithromycin resistant in 2014, still a significant increase (p<0.001) from 2011. In 2014, azithromycin resistance was identified in 5 provinces across Canada. During 2009–2012 in Canada, 5 isolates with a high level of azithromycin resistance (MIC of azithromycin ≥256 mg/L) were identified.

In 2012, seven isolates with combined decreased susceptibility to cephalosporins and resistance to azithromycin were identified (0.2%, 7/3,036). In 2013, eight (0.3%, 8/3,195) and in 2014 one (0.03%, 1/3,809) of these isolates were identified. These isolates with both decreased susceptibility to cephalosporins and resistance to azithromycin may threaten the success of the currently recommended therapy.

From 2010 to 2014 in Canada, the proportion of *N. gonorrhoeae* isolates with decreased susceptibility to cephalosporins decreased significantly. The timing of the decrease corresponded to changes in treatment guidelines from monotherapy with third-generation cephalosporins to combination therapy with ceftriaxone/azithromycin. Although causality cannot be attributed to this decline, the higher dose of ceftriaxone plus azithromycin could be more effectively treating gonococcal infections and diminishing the spread of isolates with reduced cephalosporin susceptibility. Similar data have been reported from the United Kingdom and the United States. In 2011, the United Kingdom recommended dual antimicrobial therapy with ceftriaxone (500 mg intramuscularly) and azithromycin (1 g orally in a single dose) for treatment of uncomplicated gonococcal infection (*8*). Isolates with decreased susceptibility to cefixime (MIC >0.125 mg/L) declined significantly from 10.8% in 2011 to 5.7% in 2012 and then to 5.2% in 2013 after implementation of the new guidelines (*9*). In the United States in 2012, ceftriaxone (250 mg intramuscularly) combined with azithromycin (1 g orally) or doxycycline was the recommended therapy (*10*). Decreased susceptibility to cefixime (MIC >0.25 mg/L) declined from 1.4% in 2011 to 0.4% in 2013 and decreased susceptibility to ceftriaxone (MIC >0.125 mg/L) declined from 0.4% in 2011 to 0.05% in 2013 (*11*,*12*). Although the observed decline in decreased susceptibility to cephalosporins is encouraging, during 2010–2014 in Canada, the proportion of azithromycin-resistant isolates increased significantly, to 3.3%. This increase is alarming because it approaches the 5% level at which the World Health Organization recommends reviewing and modifying national guidelines for treatment of sexually transmitted infections (*2*). Azithromycin resistance levels in Canadian isolates were higher than that reported in the United States (0.5%, 0.3%, and 0.4% in 2010, 2011, 2012, respectively) (*12*) and the United Kingdom (0.8% in 2012 and 1.6% in 2013 [MIC≥1 mg/L]) (*9*) but similar to resistance levels in Australia (2.1% in 2013) (*13*).

Limitations of this study include the representativeness of isolates, because ≈70% of gonococcal infections in Canada are diagnosed by nucleic acid amplification tests (*14*), and the current passive surveillance system collects predominately resistant isolates from provinces with different susceptibility testing methods. The proportion of resistance could be higher than that indicated by our numbers.

## Conclusions

Continued surveillance of gonococcal antimicrobial susceptibilities is vital to inform treatment guidelines and mitigate the spread of isolates with decreased susceptibility to cephalosporins and resistance to azithromycin. The data we present further support efforts to limit the spread of *N. gonorrhoeae* antimicrobial drug resistance and prevent the emergence of untreatable multidrug-resistant gonorrhea.
